# Microbial synthesis of poly-γ-glutamic acid: current progress, challenges, and future perspectives

**DOI:** 10.1186/s13068-016-0537-7

**Published:** 2016-06-29

**Authors:** Zhiting Luo, Yuan Guo, Jidong Liu, Hua Qiu, Mouming Zhao, Wei Zou, Shubo Li

**Affiliations:** College of Light Industry and Food Engineering, Guangxi University, Nanning, 530004 China; National Engineering Research Center for Non-Food Biorefinery, Guangxi Academy of Sciences, Nanning, 530004 China; College of Bioengineering, Sichuan University of Science and Engineering, Zigong, 643000 Sichuan China

**Keywords:** Poly-γ-glutamic acid, Metabolic regulation, Microbial fermentation, Strain development, Process optimization, Industrial applications

## Abstract

Poly-γ-glutamic acid (γ-PGA) is a naturally occurring biopolymer made from repeating units of l-glutamic acid, d-glutamic acid, or both. Since some bacteria are capable of vigorous γ-PGA biosynthesis from renewable biomass, γ-PGA is considered a promising bio-based chemical and is already widely used in the food, medical, and wastewater industries due to its biodegradable, non-toxic, and non-immunogenic properties. In this review, we consider the properties, biosynthetic pathway, production strategies, and applications of γ-PGA. Microbial biosynthesis of γ-PGA and the molecular mechanisms regulating production are covered in particular detail. Genetic engineering and optimization of the growth medium, process control, and downstream processing have proved to be effective strategies for lowering the cost of production, as well as manipulating the molecular mass and conformational/enantiomeric properties that facilitate screening of competitive γ-PGA producers. Finally, future prospects of microbial γ-PGA production are discussed in light of recent progress, challenges, and trends in this field.

**Electronic supplementary material:**

The online version of this article (doi:10.1186/s13068-016-0537-7) contains supplementary material, which is available to authorized users.

## Background

Poly-γ-glutamic acid (γ-PGA) is an unusual anionic homopolyamide made from d-and l-glutamic acid units connected through amide linkages between α-amino and γ-carboxylic acid groups [1] (Additional file 1: Fig. S1). Based on the glutamate residues present, γ-PGA may be classified as γ-l-PGA (only l-glutamic acid residues), γ-d-PGA (only d-glutamic acid residues), and γ-LD-PGA (both l- and d-glutamic acid residues). At present, there exist four methods for γ-PGA production: chemical synthesis, peptide synthesis, biotransformation, and microbial fermentation [2]. Compared with other methods, microbial fermentation is the most cost-effective and has numerous advantages, including inexpensive raw materials, minimal environmental pollution, high natural product purity, and mild reaction conditions. Initially discovered in 1937 by Bruckner and co-workers as part of the capsule of *Bacillus anthracis*, γ-PGA has since been found in species from all three domains of life (archaea, bacteria, and eukaryotes) [3, 4]. Most commercial γ-PGA is currently produced via microbial fermentation from biomass.

Unlike most proteinaceous materials, γ-PGA is synthesized in a ribosome-independent manner; thus, substances that inhibit protein translation (such as chloramphenicol) have no effect on the production of γ-PGA [5]. Furthermore, due to the γ-linkage of its component glutamate residues, γ-PGA is resistant to proteases that cleave α-amino linkages [6]. More importantly, as a biodegradable, water-soluble, edible, and non-toxic biopolymer, γ-PGA and its derivatives can be used safely in a wide range of applications including as thickeners, humectants, bitterness-relieving agents, cryoprotectants, sustained release materials, drug carriers, heavy metal absorbers, and animal feed additives.

Although the microbial production of γ-PGA is well established, the cost of production, including the cost of substrates as well as process costs, remains high. Most recent research on γ-PGA production is therefore focused on optimizing growth conditions to increase yield, manipulate enantiomeric composition, and alter the molecular mass. Surprisingly, only a small number of mini reviews on the biosynthesis and applications of γ-PGA have been published to date [1, 6–9]. Therefore, in this review, we have gathered together our accumulated knowledge on the bacterial physiology and catabolism of γ-PGA, and outlined the existing biological γ-PGA production processes, placing particular emphasis on improving bacterial γ-PGA fermentation.

## Overview of γ-PGA

### Structural characteristics of γ-PGA

Generally, γ-PGA adopts five conformations; α-helix, β-sheet, helix-to-random coil transition, random coil, and enveloped aggregate. The conformation can be changed by altering environmental conditions such as pH, polymer concentration, and ionic strength [10]. For example, γ-PGA adopts a largely α-helical conformation at pH 7, but predominantly β-sheet-based conformation at higher pH [11]. The enantiomeric composition also varies and can be manipulated through the extraction process after fermentation. For example, γ-PGA containing only l or d enantiomers is soluble in ethanol, whereas γ-PGA containing equimolar amounts of l and d precipitates in ethanol [6]. Manipulating the enantiomeric composition of γ-PGA to alter its properties is therefore possible [12].

The molecular mass of γ-PGA can also influence its properties and efficacy for specific applications. Microbial-derived γ-PGA generally has a relatively high molecular weight (Mw ~10^5^–8 × 10^6^ Da), which can limit industrial applications due to high viscosity, unmanageable rheology, and difficult modification [1]. Therefore, polymers with different molecular weights may be required for different purposes, and controlling the molecular weight is of fundamental and practical importance for commercial development. Recently, medium composition, alkaline hydrolysis, ultrasonic degradation, and microbial or enzymatic degradation have all been used to alter the molecular weight of γ-PGA [1]. Of these, ultrasonic irradiation provides an interesting alternative to enzymatic hydrolysis and has been proposed to reduce both the molecular weight and polydispersity of γ-PGA without disturbing the chemical composition of the polymer [13].

### Physiological function of γ-PGA

As present, the physiological function of γ-PGA is not completely understood and is believed to depend on the environment in which the organism inhabits, and whether it is bound to peptidoglycan [7]. Peptidoglycan-bound γ-PGA may protect bacterial cells against phage infections and prevent antibodies from gaining access to the bacterium [14]. *Staphylococcus epidermidis* synthesizes surface-associated γ-PGA to protect against antimicrobial peptides and escape phagocytosis, which contributes to virulence [15]. More importantly, γ-PGA can be released into the environment to sequester toxic metal ions, decrease salt concentration [4], provide a carbon source [15], and protect against adverse conditions [16]. γ-PGA can also improve the formation of biofilms and assist absorption of essential nutrients from the environment [17].

## Microbial biosynthesis of γ-PGA

**Fig. 1:**
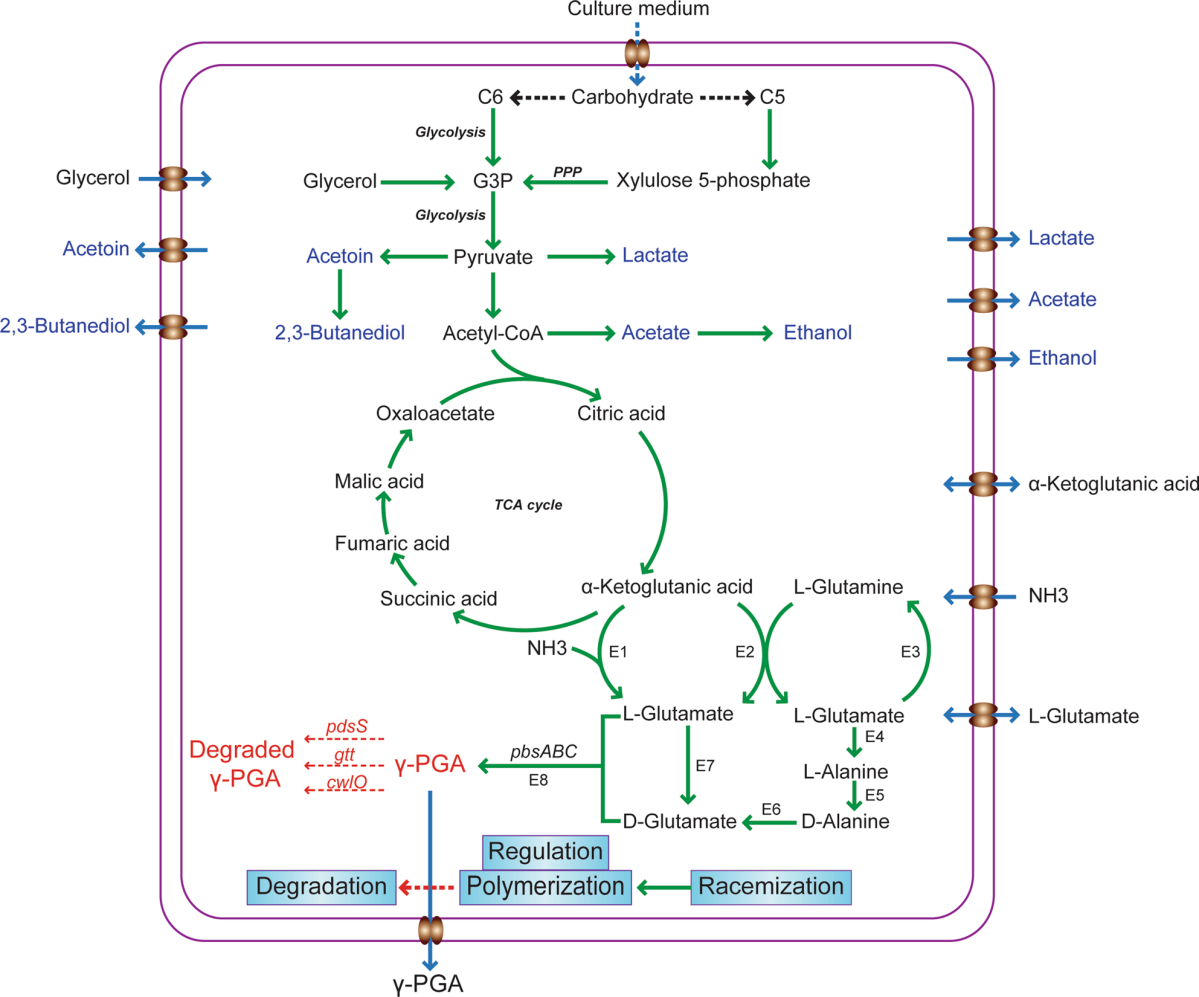
Microbial biosynthesis of γ-PGA [8, 10]. Types of substrates in the culture medium were mostly a variety of biomass materials, cane molasses, agro-industrial wastes, which could be degraded into C6 and C5 compound, entering into the main carbon metabolism via glycolysis and pentose phosphate pathway. In addition, glycerol as well as metabolic intermediates of citrate cycle was also used as candidate substrate [79]. The main byproducts were acetoin and 2,3-butanediol; other byproducts with little production were lactate, ethanol, and acetate [80]. *PPP* pentose phosphate pathway, *G3P* glyceraldehyde 3-phosphate, *E1* glutamate dehydrogenase (GD), *E2* glutamate 2-oxoglutarate aminotransferase, *E3* glutamine synthetase (GS), *E4* l-glutamic acid: pyruvate aminotransferase, *E5* alanine racemase, *E6* d-glutamic acid: pyruvate aminotransferase, *E7* direction conversion, *E8* PGA synthetase

Recently, information about the genes and enzymes involved in γ-PGA synthesis has been reported and has contributed to the design of production systems [6, 8]. As shown in Fig. 1, the proposed microbial biosynthetic pathway of γ-PGA involves l-glutamic acid units derived exogenously or endogenously (using α-ketoglutaric as a direct precursor) [18]. Biosynthesis can be divided into four distinct stages; racemization, polymerization, regulation, and degradation.

### γ-PGA racemization

Generally, γ-PGA is synthesized from d- or l-glutamate alone, or from both l and d enantiomers together [19, 20]. However, to incorporate d-glutamate into the growing l-chain, l-glutamate (exogenous or endogenous) is first converted into d-glutamate by a racemization reaction. In *B. subtilis*, two homologs of the glutamate racemase gene (*rac*E/*glr* and *yrp*C) have been identified, and *glr* is essential for converting l-glutamate into d-glutamate for the synthesis of γ-PGA [21]. Interestingly, RacE and yrpC are cytosolic enzymes with a high selectivity for glutamate and a preference for the l-form, but neither are responsible for the synthesis of γ-PGA [22]. The functions of these enzymes remains unknown [22, 23].

### γ-PGA polymerization

**Fig. 2:**
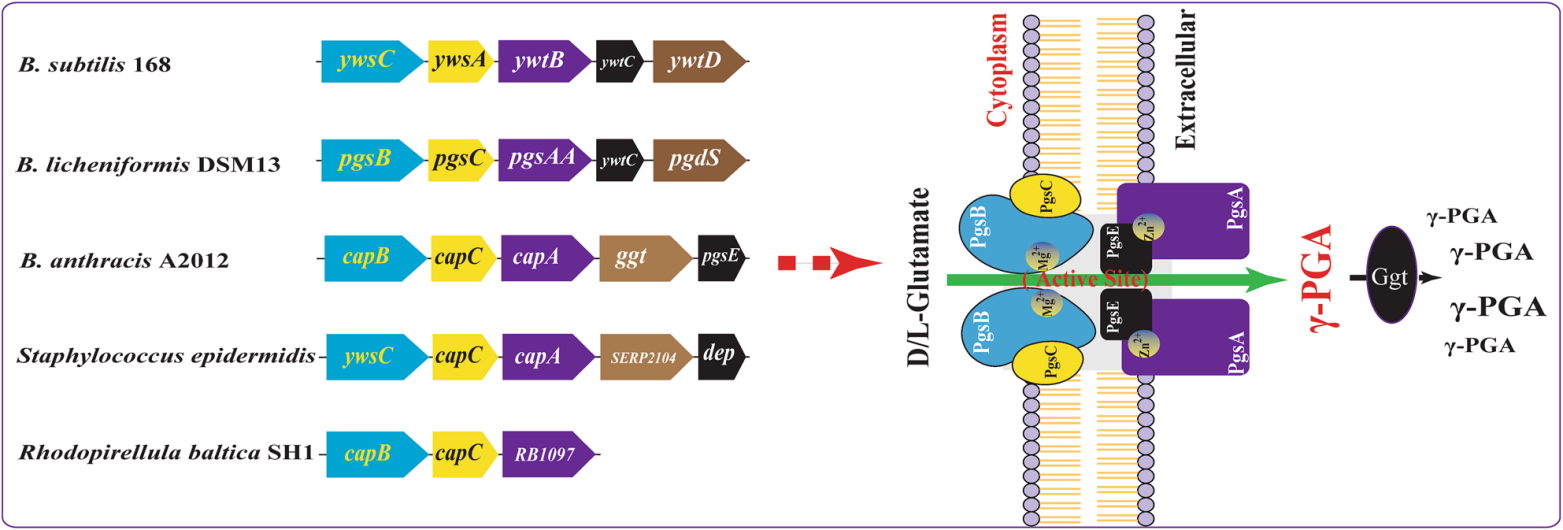
Arrangement of genes encoding γ-PGA synthetase and γ-PGA peptidase complexes in various species. All components of γ-PGA synthetase are essentially membrane associated) [8]

As shown in Fig. 2, polyglutamate synthase (pgs) is encoded by four genes (*pgs*B, C, A, and E) and their homologs in *Bacillus* species are *yws*C, *ywt*AB, and *cap*BCA [1, 24]. Recently, pgsBCA was identified as the sole machinery responsible for polymerizing γ-PGA at the active site of the synthase complex (PgsBCA) in an ATP-dependent reaction [25]. PgsB and PgsC form the main parts of the catalytic site, whereas PgsA removes the elongated chain from the active site, which is necessary for addition of the next monomer and transporting γ-PGA through the compact cell membrane [8]. The role of pgsE in the production of γ-PGA was found to be dispensable, and high concentrations of pgsB, pgsC, and pgsA were able to form γ-PGA in the absence of pgsE [26]. However, other researchers found that pgsE was essential for γ-PGA production in the presence of Zn^2+^ in *B. subtilis* [27]. This may be because the unique membrane-bound PgsBCA complex is highly unstable and hydrophobic, which could affect its isolation [7].

### γ-PGA regulation

γ-PGA synthesis is regulated by two signal transduction systems: the ComP-ComA regulator, and the two-part DegS-DegU, DegQ, and SwrA system [28]. The role of DegQ has been thoroughly investigated, and alteration of *deg*Q prevents the synthesis of γ-PGA and effectively downregulates the production of degradation enzymes [29]. However, the relationship between SwrA and DegU remains poorly understood. Osera et al. discovered that the presence of both SwrA and phosphorylated DegU (DegU-P) could fully activate the pgs operon for γ-PGA production, but the effect of either gene on both pgs transcription and γ-PGA production was negligible [30]. In contrast, Ohsawa et al. showed that a high level of DegU-P could directly activate pgs expression for γ-PGA production in place of swrA [31]. Overall, DegSU, DegQ, and ComPA appear to be involved in transcriptional regulation in response to quorum sensing, osmolarity, and phase variation signals, while SwrA appears to act at a post-transcriptional level [32].

### γ-PGA degradation

There are two enzymes capable of degrading γ-PGA in *Bacilli*: endo-γ-glutamyl peptidase and exo-γ-glutamyl peptidase [33]. Endo-γ-glutamyl peptidase can be secreted into the medium by *B. subtilis* and *B. licheniformis*, where it is able to cleave high molecular weight γ-PGA into fragments of 1000 Da to 20 kDa, which decreases dispersity as a function of depolymerization time [22, 34, 35]. In *B. subtilis*, the genes encoding endo-γ-glutamyl peptidase (*ywt*D, *dep*, or *pgd*S) are located directly downstream of, and in the same orientation as, the pgsBCA operon (Fig. 2), and the protein product includes a hydrophobic cluster (^10^F-L–L-V-A-V-I-I-C-F-L-V-P-I-M^24^) and a cleavage site (^30^A-E-A^32^) proximal to the N-terminus, indicating that the mature enzyme is secreted into the medium [36].

Exo-γ-glutamyl peptidase (Ggt) is a key enzyme in glutathione metabolism, and catalyzes the formation of γ-glutamic acid di- and tripeptides in vitro, but does not appear to be involved in γ-PGA synthesis in vivo [36, 37]. For example, ggt (or capD) was required for covalently anchoring the γ-PGA capsule to the peptidoglycan layer of the cell surface in *B. anthracis,* but not for γ-PGA synthesis [26]. As a member of the γ-glutamyl transpeptidase (GGT) family, CapD is able to cleave and subsequently transfer γ-PGA to an acceptor molecule or H_2_O, resulting in transpeptidation or hydrolysis, respectively [38]. GTTs display exohydrolase activity toward γ-PGA, releasing glutamate as a source of carbon and nitrogen [39]. In *B. subtilis*, ggt and capD are located on the chromosome distant from the pgsBCA cluster and expressed during the stationary phase under the control of the ComQXPA quorum-sensing system, but are located on a plasmid directly downstream from the pgsBCA cluster in *B. anthracis* [40].

As mentioned above, γ-PGA can be anchored to the bacterial surface or released into the medium, and CapD catalyzes the anchorage of γ-PGA to the peptidoglycan, whereas PgsS catalyzes its release. Therefore, inhibiting or knocking down γ-PGA hydrolase can result in the production of high molecular weight γ-PGA [41]. Indeed, *B. subtilis* strains deficient in exopeptidase are unable to cleave γ-PGA into fragments smaller than 10^5^ kDa, and they sporulate earlier than wild-type strains [22].

## Fermentation engineering for γ-PGA production

At present, γ-PGA can be synthesized by *Bacillus* species, *Fusobacterium nucleatum*, and some archaea and eukaryotes [3], but *Bacillus* species are used most widely to study biological γ-PGA production. Bacteria are either l-glutamate-dependent (*B. subtilis* CGMCC 0833 [42], *B. licheniformis* P-104 [43]) or non-l-glutamate-dependent (e.g. *B. subtilis* C1 [44] and *B. amyloliquefaciens* LL3 [45]) producers of γ-PGA. For l-glutamic acid-dependent bacteria, PGA yield can be enhanced by increasing the l-glutamate concentration, but this increases the cost of production significantly [8]. In contrast, due to the low cost of production and simple fermentation process, l-glutamate-independent producers are more desirable for industrial γ-PGA production, but are limited by their lower γ-PGA productivity [45]. Therefore, the cost of production (including both productivity and substrates) is a major limitation for microbial γ-PGA production.

To this end, most research on γ-PGA fermentation has focused on optimizing growth conditions to improve γ-PGA yield, alter the enantiomeric composition, and manipulate the molecular mass of γ-PGA [25]. Additionally, genetic engineering of non-glutamate-dependent producers such as *B. amyloliquefaciens* [46], *B. subtilis* [47], and *E. coli* [48] has also been used to increase γ-PGA production.

### Strain screening and improvement

Numerous *Bacillus* species have been established as γ-PGA producers, and native strains can produce more than 20 g/L of γ-PGA in fermentation processes. As shown in Table 1, the top ten strains are all rod-shaped, Gram-positive, endospore-forming members of the order *Bacillales*. Most γ-PGA producers can therefore be divided into two groups: Group I = *Bacillus* species; Group II = other bacteria.

**Table 1:** Strains, fermentation media, and control methods of the ten highest-yielding γ-GPA fermentation processes

Starting sources	Isolation or improvement methods	Fermentation medium components	Bioreactor and process control^a^	Yield (g/L)	Ref.
*B. subtilis* ZJU-7	Isolated from fermented bean curd	Glucose, l-glutamate, yeast extract, NaCl, CaCl_2_, MgSO_4_, MnSO_4_	10-L bioreactor, 300–800 rpm with 1.5 vvm, pH 6.5, 37 °C	101.1	[49]
*B. subtilis* NX-2	Using co-fermentation strategy	Glutamate, (NH4)_2_SO_4_, K_2_HPO_4_, MgSO_4_, MnSO_4_, and hydrolysis of rice straw	7.5-L bioreactor, 400 rpm with 1.2 vvm, initial pH 7.0, 32 °C	73.0	[50]
*B. subtilis* NX-2	Isolated from soil samples	Glucose, glutamate, (NH_4_)_2_SO_4_, K_2_HPO_4_, MgSO_4_, MnSO_4_	7.5-L APFB for immobilized fermentation, 32 °C, pH 7.0	71.21	[42]
*B. subtilis* MJ80	Isolated from soil samples	Glutamic acid, starch, urea, citric acid, glycerol, NaCl, K_2_HPO_4_, MgSO_4_, MnSO_4_	3-L fermenter, 37 °C, 150 rpm with 1 vvm, initial pH 7.0	68.7	[56]
*B. subtilis NX*-*2*	Isolated from soil samples	Cane molasses and monosodium glutamate waste liquor	7.5-L bioreactor, 400 rpm at 1.2 vvm, 32 °C, pH 7.0	52.1	[55]
*B. licheniformis* P-104	Isolated from Chinese soybean paste	Glucose, sodium glutamate, sodium citrate, (NH4)_2_SO_4_, MnSO_4_, MgSO_4_, K_2_HPO_4_	7-L bioreactor, 500 rpm with 1.5 vvm, 37 °C, pH 7.0, fed-batch	41.6	[43]
*B. licheniformis* NCIM 2324	Addition of metabolic precursors	Glycerol, l-glutamic acid, citric acid, (NH4)_2_SO_4_, K_2_HPO_4_, MgSO_4_, MnSO_4_	250-mL flask, 200 rpm, 37 °C, initial pH 6.5	35.75	[81]
*B. methylotrophicus* SK19.001	Isolated from soil samples	Glucose, yeast extracts, MgSO_4_, K_2_HPO_4_, MnSO_4_	250 mL flask, 200 rpm, initial pH 7.2, 37 °C	35.34	[51]
*B. subtillis* HB-1	Isolated from soil samples	Glutamate, yeast extract, NaCl, MgSO_4_, xylose, or corncob fibers hydrolysate	10-L bioreactor, 500 rpm, 37 °C, initial pH 6.5, fed-batch	28.15	[82]
*B. licheniformis* TISTR 1010	Using different feeding strategies	Glucose, citric acid, NH_4_Cl, K_2_HPO_4_, MgSO_4_, CaCl_2_, MnSO_4_, NaCl, Tween-80,	7-L fermenter, 300 rpm and 1 vvm, initial pH 7.4, 37 °C	27.5	[83]

^a^ *vvm* volumes of air per volume of broth, *APFB* aerobic plant fibrous-bed bioreactor

*Bacillus subtilis* is a Gram-positive, endospore-forming, rod-shaped bacteria that has generally been recognized as having a safe (GRAS) status and can therefore be used to produce enzymes such as alpha amylase and proteases that are used in the food and medicine industries. Isolation of *B. subtilis* strains with excellent γ-PGA production abilities has been achieved due to its ubiquitous and sporulating nature. As shown in Table 1, many *B. subtilis* strains have been widely used for producing γ-PGA, and *B. subtilis* CGMCC 1250 produces 101.1 g/L γ-PGA, demonstrating the potential this organism has for γ-PGA production [49]. More importantly, simple enrichment and screening procedures without mutagenesis or genetic manipulation identified native strains that can produce more than 20 g/L of γ-PGA [50]. *Bacillus licheniformis,* Gram-positive, endospore-forming bacterium, shares many similarities with *B. subtilis,* and this non-pathogenic organism has also been exploited for the production of γ-PGA.

Other than the two *Bacillus* species discussed above, *Bacillus methylotrophicus* SK19.001 should also be noted, because it yields a high level of γ-PGA with an ultrahigh molecular weight [51]. Other species such as *B. anthracis* and *Bacillus thuringiensis* also have the capacity for γ-PGA production [52], but these organisms attach γ-PGA to peptidoglycan instead of secreting it into the medium, making the recovery and purification procedure more difficult. More importantly, the production of γ-PGA using *B. anthracis* is not viable owing to its toxicity [53].

### Biosynthesis of γ-PGA in different hosts

With the development of metabolic engineering, homologous hosts have been engineered for γ-PGA production (Table 2). However, while much laborious manipulation has been attempted on various strains, only a low γ-PGA yield has been achieved. Therefore, only a limited number of strains are considered useful for industrial γ-PGA bioproduction, and the selection of a good strain for further improvement is the crucial starting element.

**Table 2:** Exemplar engineering of homologous and heterogeneous hosts

Strains	Engineering methods	Fermentation medium	Production (g/L)	Ref.
*B. licheniformis* WX-02	Expression of *glr* gene encoding glutamate racemase	Glucose, l-glutamic acid, sodium citrate, NH_4_Cl, MgSO_4_, K_2_HPO_4_, CaCl_2_, ZnSO_4_, MnSO_4_	14.38	[75]
*B. amyloliquefaciens* LL3	Double-deletion of genes *pgdS* and *cwlO*	Sucrose, (NH_4_)_2_SO_4_, MgSO_4_, KH_2_PO_4_, K_2_HPO_4_	7.12	[84]
*B. amyloliquefaciens* LL3	Deletion of genes (*rocR*, *rocG, gudB, odhA*)	Sucrose, (NH_4_)_2_SO_4_, MgSO_4_, KH_2_PO_4_, K_2_HPO_4_,	5.68	[85]
*B. subtilis* ISW1214	Bearing the plasmid-borne PGA synthetic system	Sucrose, NaCl, MgSO_4_, KH_2_PO_4_, NaHPO_4_,xylose	9.0	[47]
*E. coli* BL21	Cloning and overexpressing γ-PGA biosynthesis genes	Glucose, yeast extract, l-glutamic acid, (NH_4_)_2_SO_4_	3.7	[76]
*E. coli* JM 109	Co-expressing γ-PGA synthetase and glutamate racemase	LB medium supplemented with l-glutamate or glucose	0.65	[48]
*B. amyloliquefaciens*	Deletions of genes (*epsA-O*, *sac*, *lps*, *pta*, *pgdS*, *cwlO*, *luxS*, and *rocG* gene, expression of synthetic small regulatory RNAs which repressed the rocG and glnA gene	Sucrose, (NH_4_)_2_SO_4_, MgSO_4_, KH_2_PO_4_, K_2_HPO_4_	20.3	[86]
*B. subtilis PB5249*	Knockout of genes (*pgdS* and *ggt*)	l-glutamic acid, citric acid, glucose, NH_4_Cl, K_2_HPO_4_, MgSO_4_·7H_2_O, FeCl_3_·6H_2_O, CaCl_2_·2H_2_O, MnSO_4_·H_2_O, pH 6.5	40	[87]
*Bacillus licheniformis WX*-*02*	Enhanced expression of *pgdS* gene	Glucose, sodium glutamate, sodium citrate, NH_4_Cl, MgSO_4_, K_2_HPO_4_, CaCl_2_, ZnSO_4_, MnSO_4_	20.16	[88]
*Corynebacterium glutamicum*	Cloning and expressing γ-PGA biosynthesis genes	Glucose, (NH_4_)_2_SO_4_, KH_2_PO_4_, MgSO_4_·7H_2_O, FeSO_4_·7H_2_O, MnSO_4_·4H_2_O, soy protein hydrolysate, thiamine hydrochloride, CaCO_3_	18	[54]

Despite some progress, γ-PGA production remains low in these strains

Expression of γ-PGA-producing genes in heterologous hosts has been attempted (Table 2). *Escherichia coli* is the most commonly used host for γ-PGA biosynthesis, and the γ-PGA synthase genes *pgs*BCA and *rac*E from *B. licheniformis* NK-03 and *B. amyloliquefaciens* LL3 were, respectively, cloned and co-expressed in *E. coli* JM109 to evaluate γ-PGA production [48]. The engineered strain could produce γ-PGA from both glucose and l-glutamate, and co-expression of the *rac*E gene further increased the production of γ-PGA to 0.65 g/L. Another similar study was carried out using *Corynebacterium glutamicum* as the host, clone, and expression of the γ-PGA synthase genes *pgs*BCA from *Bacillus subtilis* TKPG011. The production of γ-PGA reached 18 g/L when the combinant was cultured with the limitation of biotin [54]. Those studies suggested that the selection of the appropriate γ-PGA-producing genes from the appropriate species may be one of the key issues. In any case, the final yield of γ-PGA is still far below that produced by native strains.

### Optimization of the growth medium

As shown in Fig. 1, pyruvate is the precursor for γ-GPA in many bacterial species, and its secretion is tightly associated with cell growth. Therefore, suitable culture media could support vigorous cell growth and hence generate enough precursor for γ-GPA synthesis.

Other than glucose which is the most successful carbon substrate for γ-GPA production from a variety of biomass materials, cane molasses, xylose, agro-industrial wastes, rapeseed meal, soybean residue, fructose, corncob fibers, hydrolysate, and crude glycerol have also been tested (Tables 1, 2). Although some of these substrates resulted in a modest γ-GPA yield, a wider substrate spectrum should be investigated. Cane molasses were shown to be a suitable fermentable substrate for γ-PGA production, and statistical optimization of medium components resulted in the production of 52.1 g/L of γ-PGA from cane molasses, without optimizing the fermentation process [55]. Cane molasses may provide an even higher γ-GPA yield following optimization of the strain and fermentation process.

Additionally, much work has been carried out on the nutritional requirements for cell growth to improve γ-PGA productivity and modify the D/L composition of the polymer. For an exogenous glutamate-independent producer, yeast extract proved to be an excellent nitrogen source for bacterial cell growth and γ-PGA production, but the high cost is a barrier to commercial production [51]. Therefore, attempts have been made to reduce the dosage or replace it with other media supplements such as (NH_4_)_2_SO_4_ or NH_4_Cl [56] (Table 1). As well as carbon and nitrogen sources, inorganic salts can affect the production, productivity, and quality of γ-PGA. Mn^2+^ in particular can improve cell growth, prolong cell viability, and assist the utilization of different carbon sources, as well as significantly alter the stereochemical and enantiomeric composition of γ-PGA, and increase γ-PGA production [1, 19].

### Process control

Efficient and effective control of fermentation depends on an understanding of the key biological and chemical parameters [57], and dissolved oxygen and culture pH are fundamental parameters that need careful control.

Oxygen is essential in aerobic fermentation and affects cell growth, carbon source utilization, biosynthesis of products, and NAD(P)H recycling [58]. Various strategies have been deployed to maintain oxygen supply, including the separated or combined use of oxygen-enriched air, modified impeller design, and addition of other oxygen vectors. However, for production of highly viscous biopolymers such as γ-PGA, it might be more economical and effective to replace gaseous oxygen with another molecular electron acceptor (Table 3). For example, the effects of different oxygen vectors on the synthesis and molecular weight of γ-PGA were investigated in a *B. subtilis* batch fermentation process, and 0.3 % *n*-heptane increased to 39.4 g/L and molecular weight 19.0 × 10^5^ Da [59].

**Table 3:** Application of different strategies for improving γ-PGA production

Starting sources	Fermentation strategies	Main results	Ref.
*B. licheniformis* NCIM 2324	Optimization via one factor at a time	γ-PGA production increased from 5.27 to 26.12 g/L	[89]
*B. subtilis* CGMCC 0833	Applying pH-shift control strategy	Glutamate utilization increased from 24.3 to 29.5 g/L; γ-PGA production increased from 22.2 to 27.7 g/L	[62]
*B. subtilis* NX-2	Using a two-stage strategy for agitation speed control	The concentration of γ-PGA reached 40.5 g/L with increases of 17.7 %	[90]
*B. subtilis* NX-2	Adding different oxygen vectors	The concentration of γ-PGA reached 39.4 g/L with increase of 25.1 %	[59]
*B. subtilis* BL53	Adding some precursors	The production of γ-PGA increased to 25.2 g/L	[91]
*B. subtilis* C10	Addition of organic acid	The concentration of γ-PGA increased from 17.3 to 27.7 g/L	[92]
*B. licheniformis* NCIM 2324	Addition of metabolic precursors	The concentration of γ-PGA reached 35.75 from 26.12 g/L	[81]
*B. licheniformis* TISTR 1010	Using different feeding strategies	The γ-PGA concentration reached 27.5 g/L with increase of fivefold	[83]
*B. subtilis* NX-2	Using co-fermentation strategy	The production and productivity of γ-PGA reached 73.0 g/L and 0.81 g/L/h, respectively	[50]
*B. subtilis* CCTCC M 2012347	Solid-state fermentation	Using soybean residue and cane molasses to produce 103.5 g/kg of γ-PGA at 24 h under non-sterilized condition	[93]
*B. amyloliquefaciens* C1	Solid-state fermentation	Producing 0.0437 g γ-PGA per gram of substrates at 48 h	[94]
*B. subtilis* GXA-28	Addition of KCl	The γ-PGA yield increased from 18.36 to 25.62 g/L by 39.5 %	[95]
*B. licheniformis*	Heat stress and alkaline stress treatment	The maximum γ-PGA yield reached 29.34 g/L, 185 % higher than the control	[78]
*B. licheniformis* WX-02	Alkaline pH stress treatment	The maximum γ-PGA yield reached 36.26 g/L in the 50 L bioreactor, increased by 79 % compared with the control	[96]
*B. subtilis* NX-2	Adding hydrogen peroxide	The maximum concentration of 33.9 g/L γ-PGA was obtained by adding 100 µM H_2_O_2_ to the medium after 24 h. This concentration was 20.6 % higher than that of the control	[97]

Culture pH is another important environmental factor in γ-PGA fermentation [60]. A pH of 6.5 supported rapid cell growth and high γ-PGA production in *B. licheniformis* ATCC 9945A [58], whereas the highest biomass and γ-PGA yield were achieved at pH 7 in *B. subtilis* IFO 3335 [61]. However, the optimal pH for glutamate utilization has never been taken into consideration, even though the glutamate transport system is pH sensitive and is a key factor in γ-PGA fermentation. Therefore, to further increase the utilization of glutamate and enhance the production of γ-PGA, a two-stage pH-shift control strategy was proposed and developed, in which pH was maintained at 7 for the first 24 h to obtain the maximum biomass, and then shifted to 6.5 to maximize glutamate utilization and γ-PGA production. As a result, glutamate utilization increased from 24.3 to 29.5 g/L, and consequently the yield of γ-PGA increased from 22.2 to 27.7 g/L [62].

In industrial fermentation, the choice of reactor operation mode may be vital for achieving optimal process design. A series of operation modes should be tested at small scale, such as batch, fed-batch, continuous culture, cell recycling, and cell immobilization, all of which may have their own advantages and disadvantages. For example, continuous culture can be operated at a steady state with continuous feeding, which can enhance productivity and/or lower labor intensity, but a high yield may be difficult to achieve. For γ-PGA production, batch and fed-batch are the most common fermentation strategies and, overall, the batch mode has tended to achieve a higher product yield and productivity and is the most promising method for industrial-scale γ-PGA fermentation (Table 3).

To avoid the addition of exogenous l-glutamic acid, symbiotic fermentation was also proposed and developed, in which the l-glutamate-dependent *B. subtilis* was co-cultured with *Corynebacterium glutamicum* using glucose and sucrose as a mixed carbon source. Thus, integrated bioprocesses have advantages that included shortening the fermentation time and reducing the production cost, and produced γ-PGA with an average molecular mass of 1.24 × 10^6^ Da [63].

### Product recovery

During microbial fermentation, downstream processing is always a key issue for improving process economy. As discussed above, γ-PGA fermentation is influenced by various nutritional and environmental parameters, and the effects of these variables on product recovery should be assessed. For example, excessive use of complex raw materials will pose difficulties for product isolation.

There exist three fundamentally different approaches to recovering γ-PGA from the culture broth: precipitation by complex formation, precipitation by reducing water solubility, and filtration [8]. In all cases, the first step is to remove the biomass through centrifugation or filtration with a 0.45 µm filter [64]. For complex formation, γ-PGA can be precipitated using Cu^2+^, Al^3+^, Cr^3+^, and Fe^3+^, and Cu^2+^ is the most efficient metal ion for selectively precipitating γ-PGA, even at a low concentration [16]. The resultant precipitate is re-dissolved by adding 1.5 M HCl and cleaved into monomers and oligomers. Alternatively, γ-PGA can be precipitated by reducing water solubility, following the addition of ethanol to the supernatant or filtrate and then re-dissolving in distilled water [64]. Compared with complex formation, reducing water solubility is less selective and can result in co-precipitation of proteins and polysaccharides [65]. Finally, due to the large differences in molecule size between high molecular weight γ-PGA and all other constituents of the culture broth, a series of filtration and buffer exchange steps can be applied to effectively separate γ-PGA [66]. For example, alcohol precipitation was the widely used method for the recovery of γ-PGA from cell-free broth, in which the γ-PGA recovery, concentration factor, and concentration of concentrate could reach about 80 %, 0.2, and 110 g/L, respectively, after acidification (pH 3.0) and ultrafiltration [64].

## Applications of γ-PGA

Due to being water soluble, biodegradable, edible, and non-toxic, γ-PGA and its derivatives have been applied in a broad range of industrial fields, including food, cosmetics, agriculture, medicine, and bioremediation (Table 4).

**Table 4:** Applications of γ-PGA and its derivatives

Field	Applications	Details	Ref.
Food industry	Food supplement	Promotion of absorption of bioavailable minerals, such as Ca^2+^	[68]
	Texture enhancer	Enhancing the rheological and thermal properties, and reducing the hardness of wheat bread	[98]
	Oil-reducing agent	Reducing oil uptake during deep-fat frying	[99]
	Cryoprotectant	The γ-PGA with 20 kDa could have higher antifreeze activities than high antifreeze agents like glucose	[11]
	Thickener	Enhancing viscosity for fruit juice beverage, sports drinks	[1]
	Animal feed additives	Increasing egg-shells strength; decreasing body fat, etc	[1]
Medicine	Metal chelator	Removal of heavy metals and radionuclides	[100]
	Drug carrier/deliverer	Improvement of anticancer; nanoparticle medicine	[101]
	Gene vectors	Use for gene therapy	[102]
	Tissue engineering	Possessing the better mechanical properties, such as easily removed, the more hydrophilic and cytocompatible	[103]
	Biological adhesive	Substitutes of fibrin with the better lung adhesion and air-leak sealing	[104]
Bioremediation	Biopolymer flocculant	Substitution for petro-chemically synthesized flocculants, such as polyacrylamide	[70]
	Metal chelates	Removal of heavy metals and radionuclides	[105]
	Dye removal	Effectively and circularly removing basic dyes from aqueous solution	[71]
Others	Moisturizer	Improving the qualities of skincare and hair care products	[72]
	Biocontrol agent	Increasing the nutrient consumption as well as growth of seedlings	[106]
	Biodegradable plastic	Use in biodegradable plastics with good thermoplastic property	[7]
	Antibacterial activity	Its derivatives have antibacterial activity against *Salmonella enteritidis, E. coli* and *Staphylococcus aureus*	[107]
	Functional membranes	Separation of metal ions; enantioselection of amino acids	[77]
	Protective effect	γ-PGA has a unique protective effect on phage particles	[108]

### Food industry

γ-PGA is used in the food industry, specifically in naturally occurring mucilage of *natto* (fermented soybeans), but also as a food supplement, osteoporosis-preventing agent, texture enhancer, cryoprotectant, and oil-reducing agent (Table 4). As a cryoprotectant, γ-PGA enhances the viability of probiotic bacteria during freeze-drying, and γ-PGA was found to protect *Lactobacillus paracasei* more effectively than sucrose, trehalose, or sorbitol [11, 67]. More importantly, as a food supplement, γ-PGA could effectively increase the bioavailability of calcium by increasing its solubility and intestinal absorption, which decreased bone loss in humans [68].

### Medicine

As shown in Table 2, γ-PGA and its derivatives have been exploited as metal chelators and drug carriers, and used in tissue engineering and as a biological adhesive in medicine. As a drug delivery agent, the molecular mass of γ-PGA was the decisive factor determining the drug delivery properties, including controlling the rate of drug release. For example, a γ-PGA molecular weight of ~3–6 × 10^4^ Da was used to produce paclitaxel poliglumex (a conjugate of γ-PGA and paclitaxel), and this significantly improved both the safety and efficiency of the drug (compared with standard paclitaxel) by enhancing its pharmacokinetic profile and water solubility. Furthermore, this improved tumor selectivity via enhanced accumulation and retention in tumor tissue [69].

### Wastewater treatment

Due to its non-toxic and biodegradable properties, γ-PGA offers an eco-friendly alternative for wastewater treatment. γ-PGA with a molecular weight of ~5.8–6.2 × 10^6^ Da appears to be superior to many conventional flocculants used in wastewater treatment plants operating downstream of food processing fermentation processes [70]. More interestingly, γ-PGA with a molecular weight of 9.9 × 10^5^ Da could effectively remove 98 % of basic dyes from aqueous solution at pH 1 and could then be re-used [71].

### Other applications

γ-PGA has also been explored for use in cosmetics as a hydrophilic humectant to increase the production of natural moisturizing agents such as urocanic acid, pyrrolidone carboxylic acid, and lactic acid [72]. Many other applications of γ-PGA likely remain to be discovered.

## Conclusion

During more than 70 years of γ-PGA-related research, great insight has been gained regarding its production, metabolic regulation, and applications. Owing to its biodegradability and non-toxic and non-immunogenic properties, it is used widely in the food, medicine, and wastewater industries. Biotechnological production of natural γ-PGA from renewable biomass continues to be of significant interest, especially in the face of decreasing fossil fuels and a need to reduce carbon emissions.

A lot of research has been carried out on the molecular biology (genes, enzymes, pathways) of γ-PGA and its biosynthesis in different organisms, some of which have been applied to improving its production [7, 8, 73]. The insight obtained has been used to manipulate the osmolarity to identify and isolate novel γ-PGA-producing strains from different sources [74]. Furthermore, genetic engineering of host strains has improved γ-PGA yields, expanded the substrate spectrum, and enhanced the robustness of organisms to environmental stresses to create efficient production strains [75, 76]. Advances in molecular biology have therefore helped to optimize γ-PGA production and expanded the number of uses to which γ-PGA can be applied.

The specific properties of γ-PGA determine its applications, and γ-PGA produced by different bacteria or culture conditions may therefore be suited to different uses. Optimization of the cost of production, molecular mass, and conformational/enantiomeric properties is crucial if the potential of γ-PGA is to be fully realized [75]. For instance, a greater understanding of the mechanism of passive drug targeting could lead to the rational improvement of PGA-based drug delivery systems [8]. Moreover, genetic engineering strategies such as directed evolution or site-directed mutagenesis could be used to modify the biosynthetic machinery and hence γ-PGA properties [77]. Clearly, much work remains to be done in this commercially important and academically interesting field of research.

With the increasing trend in using biomass as a carbon source for fermentation processes, much research into the biological production of γ-PGA has aimed at improving the cost-effectiveness and the efficiency of recovery. To realize better industrial production of γ-PGA from renewable biomass, further effort should be made in this area. For example, high-throughput screening of potential new producers should include thermo- and salt-tolerant bacterial extremophiles [78]. Additionally, waste biomass materials such as rice straw or manure compost from the dairy and pig industries could be exploited to lower the cost of feedstock [50]. Genetic manipulation could also be exploited to develop novel γ-PGA ‘superproducer’ strains. Finally, improving downstream γ-PGA separation processes could be decisive in improving the cost-effectiveness of production.

A greater understanding of the molecular regulatory mechanisms of γ-PGA biosynthesis and control of stereoisomers would undoubtedly prove valuable. Therefore, a systems approach that combines synthetic biology, metabolic engineering, and traditional fundamental research will likely lead to improved fermentative production of γ-PGA from renewable biomass.

## Additional file

10.1186/s13068-016-0537-7 Molecular structure of γ-PGA (chiral carbons are indicated with asterisks) [80].

## Abbreviations

γ-PGA: poly-γ-glutamic acid
γ-L-PGA: l-glutamic acid residues
γ-D-PGA: d-glutamic acid residues
γ-LD-PGA: l- and d-glutamic acid residues
Mw: molecular weight
pgs: polyglutamate synthase
GGT: γ-glutamyltranspeptidase
