# Supplementary figures and images for: Microbial synthesis of poly-γ-glutamic acid: current progress, challenges, and future perspectives
Source: Biotechnol Biofuels. 2016 Jun 29;9:134. doi: 10.1186/s13068-016-0537-7 (PMC4928254; doi:10.1186/s13068-016-0537-7)

**
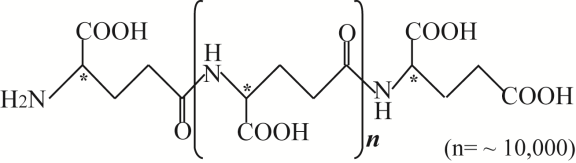
**

Fig. S1 Molecular structure of γ-PGA (chiral carbons are indicated with asterisks)[83](#_ENREF_83).

Supplement: Supplementary file 1 — 10.1186/s13068-016-0537-7 Molecular structure of γ-PGA (chiral carbons are indicated with asterisks) [80]. [file 13068_2016_537_MOESM1_ESM.docx]
